# Supplementary material for: A Systematic Literature Review of Mitochondrial DNA Analysis for Horse Genetic Diversity
Source: Animals (Basel). 2025 Mar 20;15(6):885. doi: 10.3390/ani15060885 (PMC11939364; doi:10.3390/ani15060885)
Supplement: Supplementary file 1 [file animals-15-00885-s001.zip › Total Genetics.html]

Bibliography


1.

Achilli, A.; Olivieri, A.; Soares, P.; Lancioni, H.; Kashani, B.H.; Perego, U.A.; Nergadze, S.G.; Carossa, V.; Santagostino, M.; Capomaccio, S.; et al. Mitochondrial Genomes from Modern Horses Reveal the Major Haplogroups That Underwent Domestication. *Proceedings of the National Academy of Sciences of the United States of America* **2012**, *109*, 2449–2454, doi:10.1073/pnas.1111637109.

2.

Ahmed, M.; Sheikh, A.; Mutwakil, M.; Saini, K.; Alsulaimany, F.; El Hanafy, A.; Sabir, J. Comparative Analysis of Atp6 Mitochondrial Gene Diversity in Arabian and Non-Arabian Horse Breeds. *JOURNAL OF ANIMAL AND PLANT SCIENCES* **2016**, *26*, 437–444.

3.

Almarzook, S.; Reissmann, M.; Brockmann, G.A. Diversity of Mitochondrial DNA in Three Arabian Horse Strains. *J Appl Genet* **2017**, *58*, 273–276, doi:10.1007/s13353-016-0384-z.

4.

Alvarez, I.; Fernandez, I.; Cuervo, M.; Martin, D.; Lorenzo, L.; Goyache, F. Short Communication. Mitochondrial DNA Diversity of the Founder Populations of the Asturcón Pony. *Spanish Journal of Agricultural Research* **2013**, *11*, 702–707, doi:10.5424/sjar/2013113-4127.

5.

Álvarez, I.; Fernández, I.; Lorenzo, L.; Payeras, L.; Cuervo, M.; Goyache, F. Founder and Present Maternal Diversity in Two Endangered Spanish Horse Breeds Assessed via Pedigree and Mitochondrial DNA Information. *J Anim Breed Genet* **2012**, *129*, 271–279, doi:10.1111/j.1439-0388.2012.00995.x.

6.

Alves, J.; Anjos, M.; Bastos, M.; de Oliveira, L.; Oliveira, I.; Pinto, L.; de Oliveira, C.; Costa, R.; de Camargo, G. Variability Analyses of the Maternal Lineage of Horses and Donkeys. *GENE* **2021**, *769*, doi:10.1016/j.gene.2020.145231.

7.

Bigi, D.; Perrotta, G.; Zambonelli, P. Genetic Analysis of Seven Italian Horse Breeds Based on Mitochondrial DNA D-Loop Variation. *Anim Genet* **2014**, *45*, 593–595, doi:10.1111/age.12156.

8.

Bower, M.A.; Whitten, M.; Nisbet, R.E.R.; Spencer, M.; Dominy, K.M.; Murphy, A.M.; Cassidy, R.; Barrett, E.; Hill, E.W.; Binns, M. Thoroughbred Racehorse Mitochondrial DNA Demonstrates Closer than Expected Links between Maternal Genetic History and Pedigree Records. *Journal of Animal Breeding and Genetics* **2013**, *130*, 227–235, doi:10.1111/j.1439-0388.2012.01018.x.

9.

Čačić, M.; Cubric-Curik, V.; Ristov, S.; Curik, I. Computational Approach to Utilisation of Mitochondrial DNA in the Verification of Complex Pedigree Errors. *Livestock Science* **2014**, *169*, 42–47, doi:https://doi.org/10.1016/j.livsci.2014.09.009.

10.

Cardinali, I.; Lancioni, H.; Giontella, A.; Capodiferro, M.R.; Capomaccio, S.; Buttazzoni, L.; Biggio, G.P.; Cherchi, R.; Albertini, E.; Olivieri, A.; et al. An Overview of Ten Italian Horse Breeds through Mitochondrial DNA. *PLoS ONE* **2016**, *11*, doi:10.1371/journal.pone.0153004.

11.

Castaneda, C.; Juras, R.; Khanshour, A.; Randlaht, I.; Wallner, B.; Rigler, D.; Lindgren, G.; Raudsepp, T.; Cothran, E. Population Genetic Analysis of the Estonian Native Horse Suggests Diverse and Distinct Genetics, Ancient Origin and Contribution from Unique Patrilines. *GENES* **2019**, *10*, doi:10.3390/genes10080629.

12.

Cieslak, J.; Wodas, L.; Borowska, A.; Cothran, E.G.; Khanshour, A.M.; Mackowski, M. Characterization of the Polish Primitive Horse (Konik) Maternal Lines Using Mitochondrial D-Loop Sequence Variation. *PeerJ* **2017**, *5*, e3714, doi:10.7717/peerj.3714.

13.

Cozzi, M.C.; Strillacci, M.G.; Valiati, P.; Rogliano, E.; Bagnato, A.; Longeri, M. Genetic Variability of Akhal-Teke Horses Bred in Italy. *PeerJ* **2018**, *2018*, doi:10.7717/peerj.4889.

14.

Criscione, A.; Moltisanti, V.; Chies, L.; Marletta, D.; Bordonaro, S. A Genetic Analysis of the Italian Salernitano Horse. *Animal* **2015**, *9*, 1610–1616, doi:10.1017/S1751731115001019.

15.

Csizmár, N.; Mihók, S.; Jávor, A.; Kusza, S. Genetic Analysis of the Hungarian Draft Horse Population Using Partial Mitochondrial DNA D-Loop Sequencing. *PeerJ* **2018**, *6*, e4198, doi:10.7717/peerj.4198.

16.

Czernekova, V.; Kott, T.; Majzlik, I. Mitochondrial D-Loop Sequence Variation among Hucul Horse. *CZECH JOURNAL OF ANIMAL SCIENCE* **2013**, *58*, 437–442, doi:10.17221/6992-CJAS.

17.

Dell, A.C.; Curry, M.C.; Yarnell, K.M.; Starbuck, G.R.; Wilson, P.B. Mitochondrial D-Loop Sequence Variation and Maternal Lineage in the Endangered Cleveland Bay Horse. *PLoS One* **2020**, *15*, e0243247, doi:10.1371/journal.pone.0243247.

18.

Deshpande, K.; Perez, E.; Leyva, N.; Suarez, M.; Mills, D.K. Genetic Structure of the Big Summit Herd and Neighboring Wild Horse Populations Inhabiting Herd Management Areas of Oregon. *Western North American Naturalist* **2019**, *79*, 85–98, doi:10.3398/064.079.0109.

19.

Devi, K.M.; Ahanthem, M.; Ghosh, S.K. Species Specific Mitochondrial Cytochrome c Oxidase Gene Sequence of Manipuri Pony. *INDIAN JOURNAL OF ANIMAL SCIENCES* **2014**, *84*, 1283–1288.

20.

Devi, K.M.; Ghosh, S.K. Molecular Phylogeny of Indian Horse Breeds with Special Reference to Manipuri Pony Based on Mitochondrial D-Loop. *Mol Biol Rep* **2013**, *40*, 5861–5867, doi:10.1007/s11033-013-2692-2.

21.

Dhorne-Pollet, S.; Barrey, E.; Pollet, N. A New Method for Long-Read Sequencing of Animal Mitochondrial Genomes: Application to the Identification of Equine Mitochondrial DNA Variants. *BMC Genomics* **2020**, *21*, 785, doi:10.1186/s12864-020-07183-9.

22.

Effa, K.; Rosenbom, S.; Han, J.; Dessie, T.; Beja-Pereira, A. Genetic Diversities and Historical Dynamics of Native Ethiopian Horse Populations (Equus Caballus) Inferred from Mitochondrial DNA Polymorphisms. *Genes (Basel)* **2021**, *12*, doi:10.3390/genes12020155.

23.

Engel, L.; Becker, D.; Nissen, T.; Russ, I.; Thaller, G.; Krattenmacher, N. Exploring the Origin and Relatedness of Maternal Lineages Through Analysis of Mitochondrial DNA in the Holstein Horse. *FRONTIERS IN GENETICS* **2021**, *12*, doi:10.3389/fgene.2021.632500.

24.

Engel, L.; Becker, D.; Nissen, T.; Russ, I.; Thaller, G.; Krattenmacher, N. Mitochondrial DNA Variation Contributes to the Aptitude for Dressage and Show Jumping Ability in the Holstein Horse Breed. *ANIMALS* **2022**, *12*, doi:10.3390/ani12060704.

25.

Evrigh, N.; Omri, M.; Boustan, A.; Seyedsharifi, R.; Vahedi, V. Genetic Diversity and Structure of Iranian Horses’ Population Based on Mitochondrial Markers. *JOURNAL OF EQUINE VETERINARY SCIENCE* **2018**, *64*, 107–111, doi:10.1016/j.jevs.2018.02.011.

26.

Franco, M.M.; Santos, J.B.F.; Mendonça, A.S.; Silva, T.C.F.; Antunes, R.C.; Melo, E.O. Quick Method for Identifying Horse (Equus Caballus) and Donkey (Equus Asinus) Hybrids. *Genetics and Molecular Research* **2016**, *15*, doi:10.4238/gmr.15038895.

27.

Gemingguli, M.; Iskhan, K.R.; Li, Y.; Qi, A.; Wunirifu, W.; Ding, L.Y.; Wumaierjiang, A. Genetic Diversity and Population Structure of Kazakh Horses (Equus Caballus) Inferred from mtDNA Sequences. *Genet Mol Res* **2016**, *15*, doi:10.4238/gmr.15048618.

28.

Giontella, A.; Cardinali, I.; Pieramati, C.; Cherchi, R.; Biggio, G.; Achilli, A.; Silvestrelli, M.; Lancioni, H. A Genetic Window on Sardinian Native Horse Breeds through Uniparental Molecular Systems. *ANIMALS* **2020**, *10*, doi:10.3390/ani10091544.

29.

Giontella, A.; Sarti, F.; Cardinali, I.; Giovannini, S.; Cherchi, R.; Lancioni, H.; Silvestrelli, M.; Pieramati, C. Genetic Variability and Population Structure in the Sardinian Anglo-Arab Horse. *ANIMALS* **2020**, *10*, doi:10.3390/ani10061018.

30.

Giontella, A.; Cardinali, I.; Lancioni, H.; Giovannini, S.; Pieramati, C.; Silvestrelli, M.; Sarti, F.M. Mitochondrial DNA Survey Reveals the Lack of Accuracy in Maremmano Horse Studbook Records. *Animals (Basel)* **2020**, *10*, 839, doi:10.3390/ani10050839.

31.

Glazewska, I.; Gralak, B.; Naczk, A. Differences and Changes: An Evaluation of the Genetic Diversity of Arabian Mares from Polish State Studs Born between 1996 and 2013. *ANNALS OF ANIMAL SCIENCE* **2022**, *22*, 1225–1233, doi:10.2478/aoas-2022-0033.

32.

Guo, X.; Chu, M.; Ding, X.; Pei, J.; Yan, P. The Complete Mitochondrial Genome of Chakouyi Horse (Equus Caballus). *Conservation Genetics Resources* **2017**, *9*, 173–175, doi:10.1007/s12686-016-0639-3.

33.

Guo, X.; Pei, J.; Bao, P.; Zhou, Y. Complete Mitochondrial Genome of Equus Caballus (Datong Horse). *Mitochondrial DNA. Part B. Resources* **2019**, *4*, 1223–1224, doi:10.1080/23802359.2019.1591217.

34.

Guo, X.; Pei, J.; Chu, M.; Wu, X.; Bao, P.; Ding, X.; Liang, C.; Yan, P. The Complete Mitochondrial Genome of Hequ Horse. *Mitochondrial DNA A DNA Mapp Seq Anal* **2016**, *27*, 4657–4658, doi:10.3109/19401736.2015.1106489.

35.

Hristov, P.; Yordanov, G.; Ivanova, A.; Mitkov, I.; Sirakova, D.; Mehandzyiski, I.; Radoslavov, G. Mitochondrial Diversity in Mountain Horse Population from the South-Eastern Europe. *Mitochondrial DNA A DNA Mapp Seq Anal* **2017**, *28*, 787–792, doi:10.1080/24701394.2016.1186667.

36.

Hristov, P.; Yordanov, G.; Vladov, V.; Neov, B.; Palova, N.; Radoslavov, G. Mitochondrial Profiles of the East Bulgarian and the Pleven Horse Breeds. *J Equine Vet Sci* **2020**, *88*, 102933, doi:10.1016/j.jevs.2020.102933.

37.

Hudson, W. Whole-Loop Mitochondrial DNA D-Loop Sequence Variability in Egyptian Arabian Equine Matrilines. *PLOS ONE* **2017**, *12*, doi:10.1371/journal.pone.0184309.

38.

Jimenez, L.M.; Mendez, S.; Dunner, S.; Cañón, J.; Cortés, Ó. Colombian Creole Horse Breeds: Same Origin but Different Diversity. *Genetics and Molecular Biology* **2012**, *35*, 790–796, doi:10.1590/S1415-47572012005000064.

39.

Kang, Z.; Shi, J.; Liu, T.; Zhang, Y.; Zhang, Q.; Liu, Z.; Wang, J.; Cheng, S. Genome-Wide Single-Nucleotide Polymorphism Data and Mitochondrial Hypervariable Region 1 Nucleotide Sequence Reveal the Origin of the Akhal-Teke Horse. *Anim Biosci* **2023**, *36*, 1499–1507, doi:10.5713/ab.23.0044.

40.

Khanshour, A.M.; Cothran, E.G. Maternal Phylogenetic Relationships and Genetic Variation among Arabian Horse Populations Using Whole Mitochondrial DNA D-Loop Sequencing. *BMC GENETICS* **2013**, *14*, doi:10.1186/1471-2156-14-83.

41.

Khaudov, A.D.; Duduev, A.S.; Kokov, Z.A.; Amshokov, K.K.; Zhekamukhov, M.K.; Zaitsev, A.M.; Reissmann, M. Genetic Analysis of Maternal and Paternal Lineages in Kabardian Horses by Uniparental Molecular Markers. *Open veterinary journal (Tripoli, Libya)* **2018**, *8*, 40–46, doi:10.4314/ovj.v8i1.7.

42.

Khrabrova, L.A.; Blohina, N., V.; Bazaron, B.Z.; Khamiruev, T.N. Variability of Mitochondrial DNA D-Loop Sequences in Zabaikalskaya Horse Breed. *VAVILOVSKII ZHURNAL GENETIKI I SELEKTSII* **2021**, *25*, 486–491, doi:10.18699/VJ21.055.

43.

Khrabrova, L.A.; Blohina, N.V.; Belousova, N.F.; Cothran, E.G. Estimation of the Genealogical Structure of Vyatka Horse Breed (Equus Ferus Caballus) Using DNA Analysis. *Russian Journal of Genetics* **2022**, *58*, 462–466, doi:10.1134/S1022795422040068.

44.

Khrabrova, L.A.; Nikolaeva, A.A.; Blohina, N.V.; Sorokin, S.I. Genetic Diversity of Mitochondrial DNA Haplogroups in the Don Horse Breed. *Siberian Journal of Life Sciences and Agriculture* **2023**, *15*, 278–290, doi:10.12731/2658-6649-2023-15-4-278-290.

45.

Kobayashi, I.; Akita, M.; Takasu, M.; Tozaki, T.; Kakoi, H.; Nakamura, K.; Senju, N.; Matsuyama, R.; Horii, Y. Genetic Characteristics of Feral Misaki Horses Based on Polymorphisms of Microsatellites and Mitochondrial DNA. *J Vet Med Sci* **2019**, *81*, 707–711, doi:10.1292/jvms.18-0565.

46.

KOSEMAN, A.; OZSENSOY, Y.; ERDOGAN, M.; YARALI, C.; TOPRAK, B.; ZENGIN, K.; SEKER, İ. Investigation of Genetic Structures of Coloured Horses by Mtdna D-Loop Sequence Analysis in Turkey. *Veteriner fakultesi dergisi* **2019**, *25*, 769–778, doi:10.9775/kvfd.2019.21844.

47.

Kuhl, J.; Stock, K.F.; Wulf, M.; Aurich, C. Maternal Lineage of Warmblood Mares Contributes to Variation of Gestation Length and Bias of Foal Sex Ratio. *PLOS ONE* **2015**, *10*, doi:10.1371/journal.pone.0139358.

48.

Kusza, S.; Priskin, K.; Ivankovic, A.; Jedrzejewska, B. Genetic Characterization and Population Bottleneck in the Hucul Horse Based on Microsatellite and Mitochondrial Data. *Biological journal of the Linnean Society* **2013**, *109*, 54–65, doi:10.1111/bij.12023.

49.

Kvist, L.; Niskanen, M. Modern Northern Domestic Horses Carry Mitochondrial DNA Similar to Przewalski’s Horse. *Journal of Mammalian Evolution* **2021**, *28*, 371–376, doi:10.1007/s10914-020-09517-6.

50.

Kvist, L.; Niskanen, M.; Mannermaa, K.; Wutke, S.; Aspi, J. Genetic Variability and History of a Native Finnish Horse Breed. *Genet Sel Evol* **2019**, *51*, 35, doi:10.1186/s12711-019-0480-8.

51.

Lancioni, H.; Cardinali, I.; Giontella, A.; Antognoni, M.T.; Miglio, A. Mitochondrial DNA Variation in the Italian Heavy Draught Horse. *PeerJ* **2020**, *2020*, doi:10.7717/peerj.8996.

52.

Lin, X.; Zheng, H.-X.; Davie, A.; Zhou, S.; Wen, L.; Meng, J.; Zhang, Y.; Aladaer, Q.; Liu, B.; Liu, W.-J.; et al. Association of Low Race Performance with mtDNA Haplogroup L3b of Australian Thoroughbred Horses. *MITOCHONDRIAL DNA PART A* **2018**, *29*, 323–330, doi:10.1080/24701394.2016.1278535.

53.

Liu, G.; Xu, C.-Q.; Cao, Q.; Zimmermann, W.; Songer, M.; Zhao, S.-S.; Li, K.; Hu, D.-F. Mitochondrial and Pedigree Analysis in Przewalski’s Horse Populations: Implications for Genetic Management and Reintroductions. *MITOCHONDRIAL DNA* **2014**, *25*, 313–318, doi:10.3109/19401736.2013.800487.

54.

Liu, S.; Fu, C.; Yang, Y.; Zhang, Y.; Ma, H.; Xiong, Z.; Ling, Y.; Zhao, C. Current Genetic Conservation of Chinese Indigenous Horses Revealed with Y-Chromosomal and Mitochondrial DNA Polymorphisms. *G3 (Bethesda)* **2021**, *11*, jkab008, doi:10.1093/g3journal/jkab008.

55.

Lu, H.; Bai, H.; Wang, L.; Zhang, T. Abundant Genetic Diversity and Maternal Origins of Modern Horses. *Canadian journal of animal science* **2019**, *99*, 929, doi:10.1139/cjas-2018-0201.

56.

Ma, H.; Wu, Y.; Xiang, H.; Yang, Y.; Wang, M.; Zhao, C.; Wu, C. Some Maternal Lineages of Domestic Horses May Have Origins in East Asia Revealed with Further Evidence of Mitochondrial Genomes and HVR-1 Sequences. *PEERJ* **2018**, *6*, doi:10.7717/peerj.4896.

57.

Machmoum, M.; Badaoui, B.; Petit, D.; Germot, A.; El Alaoui, M.A.; Boujenane, I.; Piro, M. Genetic Diversity and Maternal Phylogenetic Relationships among Populations and Strains of Arabian Show Horses. *Animals (Basel)* **2023**, *13*, 2021, doi:10.3390/ani13122021.

58.

Maniego, J.; Pesko, B.; Habershon-Butcher, J.; Hincks, P.; Taylor, P.; Tozaki, T.; Ohnuma, A.; Stewart, G.; Proudman, C.; Ryder, E. Use of Mitochondrial Sequencing to Detect Gene Doping in Horses via Gene Editing and Somatic Cell Nuclear Transfer. *Drug Testing and Analysis* **2022**, *14*, 1429–1437, doi:10.1002/dta.3267.

59.

Morelli, L.; Useli, A.; Sanna, D.; Barbato, M.; Contu, D.; Pala, M.; Cancedda, M.; Francalacci, P. Mitochondrial DNA Lineages of Italian Giara and Sarcidano Horses. *Genet Mol Res* **2014**, *13*, 8241–8257, doi:10.4238/2014.October.20.1.

60.

Moridi, M.; Masoudi, A.A.; Vaez Torshizi, R.; Hill, E.W. Mitochondrial DNA D-Loop Sequence Variation in Maternal Lineages of Iranian Native Horses. *Anim Genet* **2013**, *44*, 209–213, doi:10.1111/j.1365-2052.2012.02389.x.

61.

Musiał, A.D.; Ropka-Molik, K.; Stefaniuk-Szmukier, M.; Myćka, G.; Bieniek, A.; Yasynetska, N. Characteristic of Przewalski Horses Population from Askania-Nova Reserve Based on Genetic Markers. *Molecular Biology Reports* **2023**, doi:10.1007/s11033-023-08581-4.

62.

Musiał, A.D.; Radović, L.; Stefaniuk-Szmukier, M.; Bieniek, A.; Wallner, B.; Ropka-Molik, K. Mitochondrial DNA and Y Chromosome Reveal the Genetic Structure of the Native Polish Konik Horse Population. *PeerJ* **2024**, *12*, e17549, doi:10.7717/peerj.17549.

63.

Myćka, G.; Klecel, W.; Stefaniuk-Szmukier, M.; Jaworska, J.; Musiał, A.D.; Ropka-Molik, K. Mitochondrial Whole D-Loop Variability in Polish Draft Horses of Sztumski Subtype. *Animals (Basel)* **2022**, *12*, 1870, doi:10.3390/ani12151870.

64.

Nguyen, T.B.; Paul, R.C.; Okuda, Y.; Le, T.N.A.; Pham, P.T.K.; Kaissar, K.J.; Kazhmurat, A.; Bibigul, S.; Bakhtin, M.; Kazymbet, P.; et al. Genetic Characterization of Kushum Horses in Kazakhstan Based on Haplotypes of Mtdna and y Chromosome, and Genes Associated with Important Traits of the Horses. *Journal of Equine Science* **2020**, *31*, 35–43, doi:10.1294/jes.31.35.

65.

Nikbakhsh, M.; Varkoohi, S.; Seyedabadi, H.R. Mitochondrial DNA D-Loop Hyper-Variable Region 1 Variability in Kurdish Horse Breed. *Vet Med Sci* **2023**, *9*, 721–728, doi:10.1002/vms3.996.

66.

Ning, T.; Ling, Y.; Hu, S.; Ardalan, A.; Li, J.; Mitra, B.; Chaudhuri, T.; Guan, W.; Zhao, Q.; Ma, Y.; et al. Local Origin or External Input: Modern Horse Origin in East Asia. *BMC EVOLUTIONARY BIOLOGY* **2019**, *19*, doi:10.1186/s12862-019-1532-y.

67.

Novoa-Bravo, M.; Bernal-Pinilla, E.; García, L.F. Microevolution Operating in Domestic Animals: Evidence from the Colombian Paso Horses. *Mammalian Biology* **2021**, *101*, 181–192, doi:10.1007/s42991-021-00103-8.

68.

Othman, O.E.; Mahrous, K.F.; Shafey, H.I. Mitochondrial DNA Genetic Variations among Four Horse Populations in Egypt. *J Genet Eng Biotechnol* **2017**, *15*, 469–474, doi:10.1016/j.jgeb.2017.06.004.

69.

Ovchinnikov, I.V.; Dahms, T.; Herauf, B.; McCann, B.; Juras, R.; Castaneda, C.; Cothran, E.G. Genetic Diversity and Origin of the Feral Horses in Theodore Roosevelt National Park. *PLoS ONE* **2018**, *13*, doi:10.1371/journal.pone.0200795.

70.

Peng, M.; Fan, L.; Shi, N.; Ning, T.; Yao, Y.; Murphy, R.; Wang, W.; Zhang, Y. DomeTree: A Canonical Toolkit for Mitochondrial DNA Analyses in Domesticated Animals. *MOLECULAR ECOLOGY RESOURCES* **2015**, *15*, 1238–1242, doi:10.1111/1755-0998.12386.

71.

Prystupa, J.M.; Hind, P.; Cothran, E.G.; Plante, Y. Maternal Lineages in Native Canadian Equine Populations and Their Relationship to the Nordic and Mountain and Moorland Pony Breeds. *J Hered* **2012**, *103*, 380–390, doi:10.1093/jhered/ess003.

72.

Senju, N.; Tozaki, T.; Kakoi, H.; Shinjo, A.; Matsuyama, R.; Almunia, J.; Takasu, M. Genetic Diversity of the Yonaguni Horse Based on Polymorphisms in Microsatellites and Mitochondria DNA. *JOURNAL OF VETERINARY MEDICAL SCIENCE* **2017**, *79*, doi:10.1292/jvms.16-0040.

73.

Senju, N.; Tozaki, T.; Kakoi, H.; Almunia, J.; Maeda, M.; Matsuyama, R.; Takasu, M. Genetic Characterization of the Miyako Horse Based on Polymorphisms of Microsatellites and Mitochondrial DNA. *J Vet Med Sci* **2017**, *79*, 218–223, doi:10.1292/jvms.16-0111.

74.

Sharif, M.B.; Fitak, R.R.; Wallner, B.; Orozco-terWengel, P.; Frewin, S.; Fremaux, M.; Mohandesan, E. Reconstruction of the Major Maternal and Paternal Lineages in the Feral New Zealand Kaimanawa Horses. *Animals (Basel)* **2022**, *12*, 3508, doi:10.3390/ani12243508.

75.

Sheikh, A.; Ahmed, M.; Mutawakil, M.; Saini, K.; Alsulaimany, F.; El Hanafy, A.; Sabir, J. SNP Mapping and Phylogenetic Analysis of Saudi Arabian Horse Breeds Based on Mitochondrial Genome Sequencing. *INDIAN JOURNAL OF EXPERIMENTAL BIOLOGY* **2019**, *57*, 225–230.

76.

Sheikh, A. Mitochondrial DNA Sequencing of Kehilan and Hamdani Horses from Saudi Arabia. *Saudi J Biol Sci* **2023**, *30*, 103741, doi:10.1016/j.sjbs.2023.103741.

77.

Sild, E.; Värv, S.; Kaart, T.; Kantanen, J.; Popov, R.; Viinalass, H. Maternal and Paternal Genetic Variation in Estonian Local Horse Breeds in the Context of Geographically Adjacent and Distant Eurasian Breeds. *Anim Genet* **2019**, *50*, 757–760, doi:10.1111/age.12835.

78.

Sun, Y.; Jiang, Q.; Yang, C.; Wang, X.; Tian, F.; Wang, Y.; Ma, Y.; Ju, Z.; Huang, J.; Zhou, X.; et al. Characterization of Complete Mitochondrial Genome of Dezhou Donkey (Equus Asinus) and Evolutionary Analysis. *CURRENT GENETICS* **2016**, *62*, 383–390, doi:10.1007/s00294-015-0531-9.

79.

Sziszkosz, N.; Mihók, S.; Jávor, A.; Kusza, S. Genetic Diversity of the Hungarian Gidran Horse in Two Mitochondrial DNA Markers. *PEERJ* **2016**, *4*, doi:10.7717/peerj.1894.

80.

Takasu, M.; Ishihara, N.; Tozaki, T.; Kakoi, H.; Maeda, M.; Mukoyama, H. Genetic Diversity of Maternal Lineage in the Endangered Kiso Horse Based on Polymorphism of the Mitochondrial DNA D-Loop Region. *J Vet Med Sci* **2014**, *76*, 1451–1456, doi:10.1292/jvms.14-0231.

81.

Voronkova, V.; Nikolaeva, E.; Piskunov, A.; Babayan, O.; Takasu, M.; Tozaki, T.; Svishcheva, G.; Stolpovsky, Y. Assessment of Genetic Diversity and Structure of Russian and Mongolian Autochthonous Horse Breeds Using Nuclear and Mitochondrial DNA Markers. *RUSSIAN JOURNAL OF GENETICS* **2022**, *58*, 927–943, doi:10.1134/S1022795422080105.

82.

Winton, C.L.; Plante, Y.; Hind, P.; McMahon, R.; Hegarty, M.J.; McEwan, N.R.; Davies-Morel, M.C.G.; Morgan, C.M.; Powell, W.; Nash, D.M. Comparative Genetic Diversity in a Sample of Pony Breeds from the U.K. and North America: A Case Study in the Conservation of Global Genetic Resources. *Ecol Evol* **2015**, *5*, 3507–3522, doi:10.1002/ece3.1562.

83.

Wolfsberger, W.; Ayala, N.; Castro-Marquez, S.; Irizarry-Negron, V.; Potapchuk, A.; Shchubelka, K.; Potish, L.; Majeske, A.; Oliver, L.; Lameiro, A.; et al. Genetic Diversity and Selection in Puerto Rican Horses. *SCIENTIFIC REPORTS* **2022**, *12*, doi:10.1038/s41598-021-04537-5.

84.

Yang, L.; Kong, X.; Yang, S.; Dong, X.; Yang, J.; Gou, X.; Zhang, H. Haplotype Diversity in Mitochondrial DNA Reveals the Multiple Origins of Tibetan Horse. *PLOS ONE* **2018**, *13*, doi:10.1371/journal.pone.0201564.

85.

Yang, S.; Li, A.-P.; Xu, L.; Yang, H. Short Communication: Mitochondrial DNA D-Loop Sequence Diversity and Origin of Chinese Pony Breeds ( Equus Caballus ). *Canadian journal of animal science* **2013**, *93*, 313–319, doi:10.4141/cjas2012-160.

86.

Yang, Y.; Zhu, Q.; Liu, S.; Zhao, C.; Wu, C. The Origin of Chinese Domestic Horses Revealed with Novel mtDNA Variants. *ANIMAL SCIENCE JOURNAL* **2017**, *88*, 19–26, doi:10.1111/asj.12583.

87.

Yoon, S.; Kim, J.; Shin, D.; Cho, S.; Kwak, W.; Lee, H.; Park, K.; Kim, H. Complete Mitochondrial Genome Sequences of Korean Native Horse from Jeju Island: Uncovering the Spatio-Temporal Dynamics. *MOLECULAR BIOLOGY REPORTS* **2017**, *44*, 233–242, doi:10.1007/s11033-017-4101-8.

88.

Yoon, S.H.; Lee, W.; Ahn, H.; Caetano-Anolles, K.; Park, K.-D.; Kim, H. Origin and Spread of Thoroughbred Racehorses Inferred from Complete Mitochondrial Genome Sequences: Phylogenomic and Bayesian Coalescent Perspectives. *PLoS One* **2018**, *13*, e0203917, doi:10.1371/journal.pone.0203917.

89.

Yordanov, G.; Palova, N.; Mehandjyiski, I.; Hristov, P. Mitochondrial DNA Sequencing Illuminates Genetic Diversity and Origin of Hunagrian Nonius Horse Breed and His Relatives–Danubian Horse and Serbian Nonius. *Animal Biotechnology* **2023**, *34*, 3897–3907, doi:10.1080/10495398.2023.2237533.

90.

Zhang, T.; Lu, H.; Chen, C.; Jiang, H.; Wu, S. Genetic Diversity of mtDNA D-Loop and Maternal Origin of Three Chinese Native Horse Breeds. *Asian-Australas J Anim Sci* **2012**, *25*, 921–926, doi:10.5713/ajas.2011.11483.

91.

Available online: http://www.isez.pan.krakow.pl/journals/folia/pdf/72(2)/72(2)\_03.pdf (accessed on 6 March 2025).
