# Supplementary figures and images for: A Systematic Literature Review of Mitochondrial DNA Analysis for Horse Genetic Diversity
Source: Animals (Basel). 2025 Mar 20;15(6):885. doi: 10.3390/ani15060885 (PMC11939364; doi:10.3390/ani15060885)

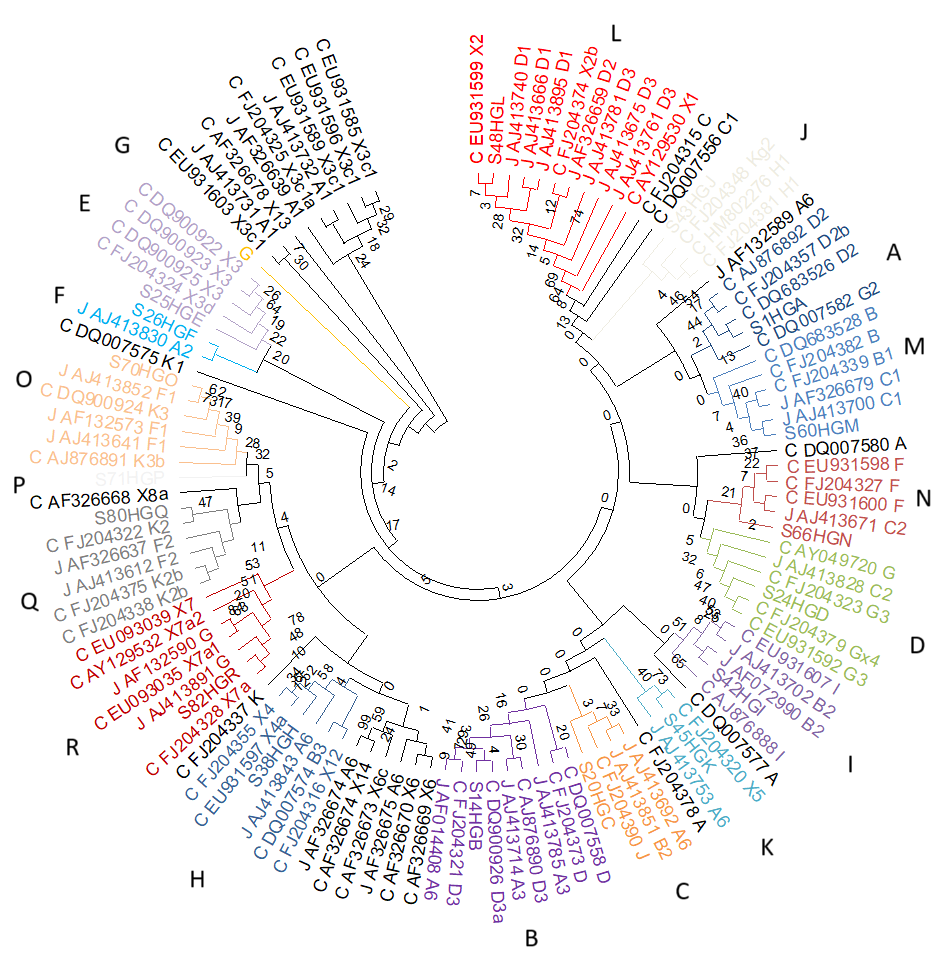

Supplement: Supplementary file 1 [file animals-15-00885-s001.zip › Figure S2.png]

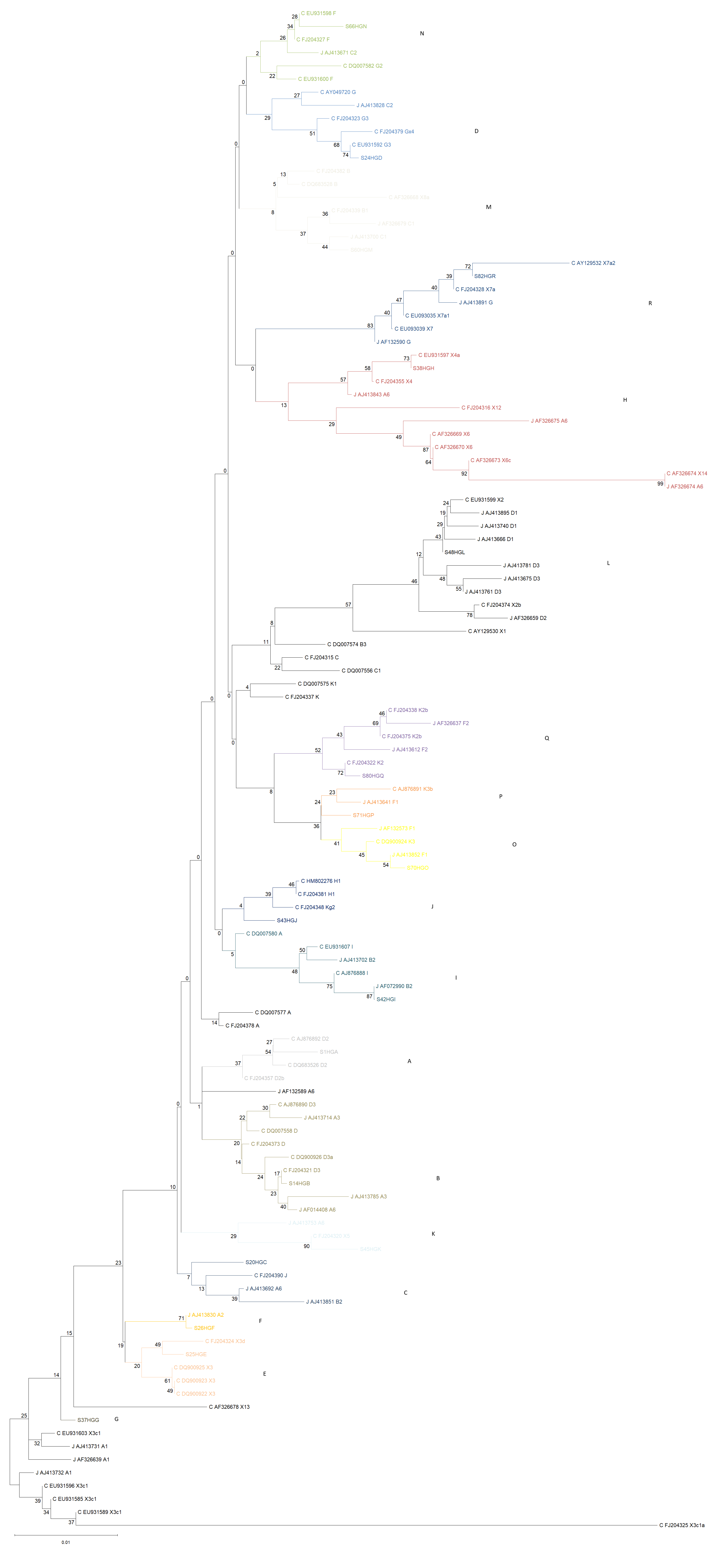

Supplement: Supplementary file 1 [file animals-15-00885-s001.zip › Figure S1.png]
